# Cutaneous Manifestations of Lyme Borreliosis in Children—A Case Series and Review

**DOI:** 10.3390/life13010072

**Published:** 2022-12-27

**Authors:** Agnieszka Myszkowska-Torz, Mateusz Tomaszewski, Michał Kotowski, Cezary Witczak, Magdalena Figlerowicz, Katarzyna Mazur-Melewska

**Affiliations:** 1 Department of Infectious Diseases and Child Neurology, Karol Marcinkowski University of Medical Sciences, 61-701 Poznań, Poland; 2 Department of Orthodontics and Temporomanidbular Disorders, Karol Marcinkowski University of Medical Sciences, 61-701 Poznań, Poland; 3 Department of Pediatric Otolaryngology, Karol Marcinkowski University of Medical Sciences, 61-701 Poznań, Poland

**Keywords:** Lyme disease, skin, children, erythema migrans, acrodermatitis chronica atrophicans, borrelial lymphocytoma, morphea

## Abstract

The occurrence of skin lesions is the earliest symptom of Lyme disease, and the diagnosis of these lesions and appropriate treatment may prevent complications of the disease, which are mainly neurological. The cutaneous presentation in borreliosis is heterogeneous. There are typical lesions that constitute the basis for the diagnosis of Lyme disease, and atypical ones, which cause significant diagnostic difficulties especially when the patient does not remember the tick bite. This study aims to describe the heterogeneous skin symptoms of Lyme borreliosis, as well as offer a practical approach for the recognition of the disease. Based on pediatric cases from clinical practice, rare cutaneous presentations of Lyme disease at various stages of illness and therapy are presented. Diagnostic recommendations for recognizing individual forms are discussed.

## 1. Introduction

There has been an increase in the incidence of tick-borne diseases in Europe in recent years, including Lyme borreliosis (LB), tick-borne encephalitis, human anaplasmosis, and babesiosis [1]. According to the latest data, the incidence of LB in Europe is highest in Germany, Austria, Slovenia, Sweden, and Poland [2,3,4]. The first cases of the disease in Poland were recorded in the mid-1980s. The main vectors of infection with spirochaetes of the species *Borrelia burgdorferi* (Bb) in Europe, including Poland, are hard ticks of the species *Ixodes ricinus*. These ticks are tri-host arachnids, and during each developmental stage they suck the blood of a different host. Larvae and nymphs usually parasitize various species of small mammals, and less often birds. Adults, in turn, feed on larger animals i.e., sheep, goats, deer, wild boars, foxes, hares, rabbits, and dogs [5]. Humans can be a host at any stage of tick development. Infection occurs when a tick penetrates the skin of the victim with the help of a hypostome. Bacteria take advantage of the immunomodulatory properties of tick saliva, thanks to which they can increase their pathogenicity and avoid the immune response from the host. Serum resistance and complement activation also play an important role in pathogenesis [6]. Infection results in the expression of lipoproteins, which in turn activate various inflammatory cells and mediators. After being introduced into the skin along with saliva, the spirochaetes lead to local infection, and then after entering blood and lymph they can cause disseminated infections in various organs. The effect of such transmission is a phased course of the disease, including an early stage with local manifestation and a late, generalized stage. In both phases, skin, joint, nervous system, and heart symptoms can be observed. The presence of skin lesions is possible in both phases of the disease, but in the case of pediatric patients, the diagnosis of a lesion that is typical of the early phase—erythema migrans (EM)—is much more frequent [7]. Other types of lesions, such as multiple erythema migrans (MEM) or borrelial lymphocytoma (BL), are less frequently observed, and therefore they are often a diagnostic problem for the general practitioner [8].

This study aims to describe the heterogeneous skin symptoms of LB, as well as offer a practical approach for the recognition of the disease.

## 2. Case Series

### 2.1. Case 1

A 4-year-old child came to the Emergency Room of the Karol Jonscher Hospital University of Medical Sciences in Poznań, Poland, with her parents because of skin lesions that had been present for 4 weeks on the skin of both legs and the lower abdomen. The lesions had occurred on both lower limbs simultaneously, and gradually spread towards the lower abdomen. The child had been born at term (first pregnancy for mother), had shown normal development to date, and had suffered only from sporadic infections of the upper respiratory tract. Six weeks prior to presentation, the patient had been bitten by a tick in the groin. The appearance of skin lesions was not accompanied by a deterioration in well-being or fever. Due to the suspicion of LB, the child was referred to the Infectious Diseases Outpatient Clinic in the Karol Jonscher Hospital University of Medical Sciences in Poznań, Poland.

**Figure 1:**
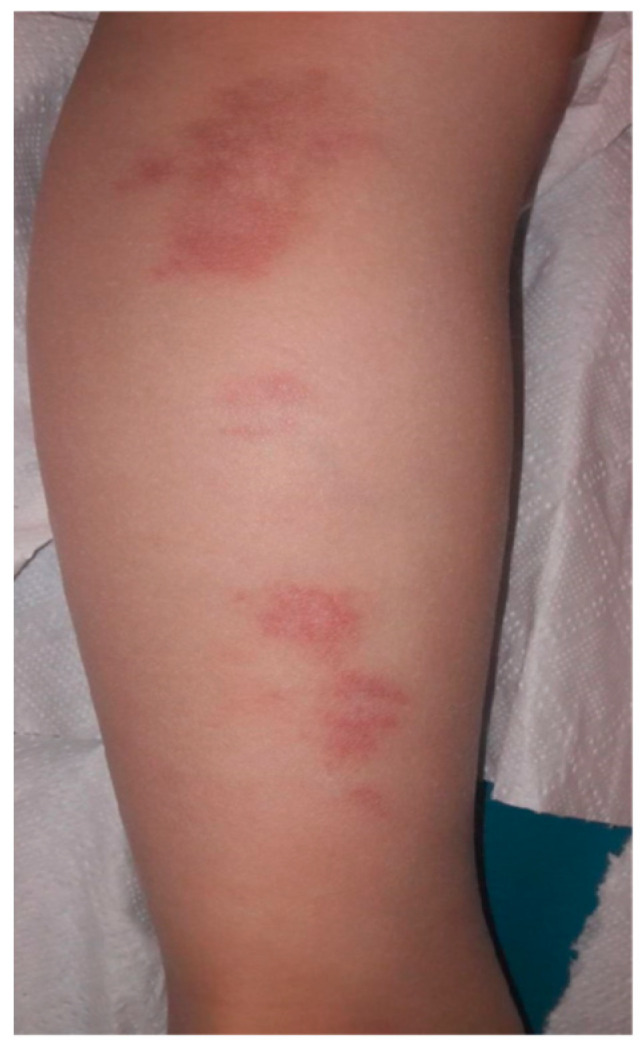
Atypical multiple erythema migrans in a 4-year-old child with Lyme borreliosis.

Upon clinical examination, the infectious diseases doctor found painful, brownish, raised lesions over the back of her legs, associated with surrounding redness that was progressively increasing (Figure 1).

Induration and tenderness were present over the lesions, and local popliteal lymphadenopathy was seen. Additionally, the patient had spots on the lower abdomen. There was no joint swelling or itching of the skin. The remaining physical examination was normal. The laboratory tests performed 5 weeks from the appearance of the first skin changes included a complete hemogram with a peripheral blood smear, which was found to be normal. The concentrations of C-reactive protein (CRP), fibrinogen, and coagulation factors were also normal. A serological test with enzyme immunoassay (ELISA) was performed for Bb: the concentrations of immunoglobulin M (IgM) and immunoglobulin G (IgG) were raised at 82.66 AU/mL and 44.3 AU/mL, respectively (cut-off for positive tests: above 22 AU/mL) (CLIA method; Liaison system; DiaSorin SpA, Saluggia, Italy). A Western blot (WB) revealed positive IgM antibodies against OspC (p25) from *Borrelia afzeli, B. burgdorferi sensu stricto* and *B. garinii* and against flagellin (p41), and positive IgG antibodies against OspC from *Borrelia afzeli*, *B. burgdorferi sensu stricto* and *B. garinii*, flagellin (p41) from *B. afzeli*, and antigen VIsE from *B. garinii* and *B. burgdorferi sensu stricto* (EUROLINE Borrelia-RN-AT; Euroimmun AG, PerkinElmer, Inc., Waltham, MA, USA). The atypical form of multiple erythema migrans (MEM) was diagnosed.

Treatment was initiated immediately with amoxicillin at a dose of 50 mg/kg body weight/day [5,9]. After 1 week of treatment, the lesions had not progressed beyond their initial size. At 2 weeks, there was a mild improvement. The erythema on the stomach had decreased, and the purple lesions on the legs were less intense and tender. The antibiotic was continued for a total of 4 weeks resulting in clinical resolution of the lesions. Pediatric evaluation of the child, performed 6 weeks after the end of therapy, showed no skin changes.

### 2.2. Case 2

An 11-year-old patient who had not been chronically ill to date was referred in March 2021 to the Infectious Diseases Outpatient Clinic in the Karol Jonscher Hospital University of Medical Sciences in Poznań, Poland, due to the suspicion of borrelial lymphocytoma. At an appointment in November 2020, the child’s parents observed ear asymmetry and enlargement of the right ear auricula. The patient consulted with their family doctor who recommended topical therapy with mupirocin ointment. Due to the lack of improvement, the parents presented the boy to the ENT specialist who referred the child to the Laryngology Department for a biopsy. The collected biopsy material revealed a fragment of the skin with abundant lymphocytic infiltration in the stroma, lymphoid cells represented by T (Cd3+) and B (Cd20+) lymphocytes, and a few polyclonal plasma cells (lambda+, kappa+). The reactive lesions were typical for lymphocytoma cutis or pseudo lymphocytoma.

**Figure 2:**
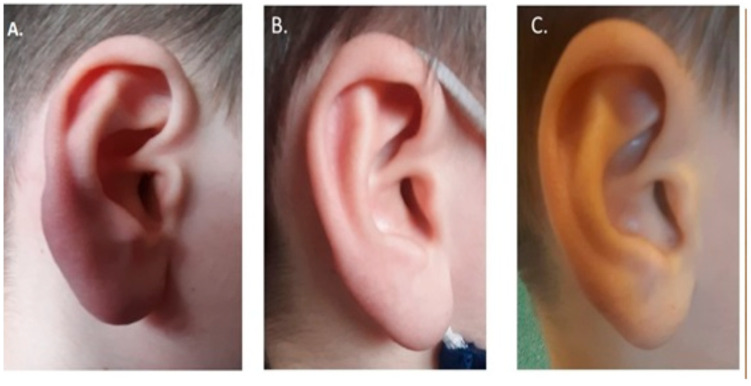
Skin lesions of the right ear of a patient with borrelial lymphocytoma—clinical picture before treatment (**A**), during treatment (**B**), and two months after the end of treatment (**C**).

During the consultation, the infectious diseases specialist noted clear asymmetry of the auricles (the right auricle was bigger), and that the right ear had a hard clear bluish-red swelling of the lobe. The lesion was painless, not shifting in relation to the substrate (Figure 2A). In addition, the skin was unchanged, without abnormal erythematous changes. Peripheral lymph nodes were not enlarged and were painless. No abnormalities were reported in the full pediatric study. A two-stage diagnosis of Lyme disease was performed (5 months from the appearance of the first skin symptoms) and confirmed with the ELISA test, with positive antibodies in the IgG class against Bb (IgG—95 AU/mL; positive result above 22 AU/mL; IgM—17 AU/mL; positive above 22 AU/mL) (CLIA method; Liaison system; DiaSorin SpA, Saluggia, Italy). The result was confirmed by Western blot (IgG positive for: p100, VIsE, p41, p39, OspC B antigens from *B. afzeli* and p18 from *B. garinii*) (EUROLINE Borrelia-RN-AT; Euroimmun AG, PerkinElmer, Inc., Waltham, MA, USA). Borrelial lymphocytoma cutis (LC) was diagnosed.

The patient was treated with oral amoxicillin at a dose of 50 mg/kg body weight/day [5,9]. The follow-up study, which was carried out in the third week of treatment, showed a clear reduction in the lesion within the ear lobe (Figure 2B). Treatment was continued for up to 28 days, and a re-check 6 weeks after antibiotic initiation showed complete resolution of the skin lesion (Figure 2C).

### 2.3. Case 3

An 8-year-old patient presented at the Infectious Diseases Outpatient Clinic in the Karol Jonscher Hospital University of Medical Sciences in Poznań, with a 7-month history of indurated plaques on the right lower leg. The child was from the mother’s first pregnancy, was born at term, had not suffered from chronic diseases to date, and had sporadically presented respiratory system infections. In December 2021, the patient developed a bluish-red, limited hardening of the skin with a diameter of more than 7 cm, accompanied by itching. The skin in the center of the lesion was thinner, with elements of atrophy. Due to the suspicion of allergy, the child was given antihistamines which had no therapeutic effect. Approximately 2 months after the onset of skin lesions, the patient consulted with a dermatologist and local treatment with aclometasone cream was started. The steroid therapy did not bring any improvement. Four months after the appearance of skin lesions, the child began to complain about pain in the joints and muscles of both legs. Despite these general complaints, the patient was not referred for any diagnostic blood tests. The child lived in the suburbs, and she often stayed in the forest because of her father’s profession as a forester. Both parents suffered from chronic Lyme disease.

**Figure 3:**
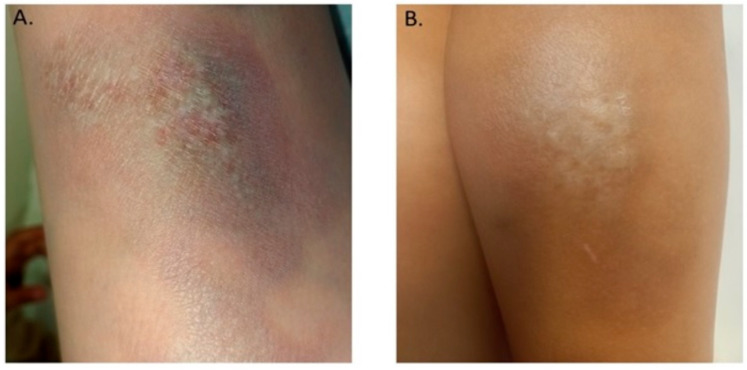
Lesions on the skin of the right lower leg of a patient with Morphea—clinical picture before treatment (**A**) and one month after the end of antibiotic therapy (**B**).

During the consultation, the infectious diseases specialist noted diffuse, atrophic, hypopigmented, indurated plaques located on the left lower leg. Epidermal defects were visible in the central areas of sclerosis (Figure 3A).

The serological diagnostics revealed an elevated concentration of Bb antibodies in the ELISA test (IgM—26.44 AU/mL; IgG—78.01 AU/mL; positive result above 22 AU/mL) (CLIA method; Liaison system; DiaSorin SpA, Saluggia, Italy). The WB was also positive for both IgM and IgG (positive antibodies against OspC (p25) from *B. afzeli*, *B. burgdorferi sensu stricto* and *B. garinii* and for antibodies against flagellin (p41) from *B. afzeli*; and for antibodies to VIsE antigen from *B. garinii*, *B. burgdorferi* sensu stricto, and *B. afzeli* in the IgG class) (EUROLINE Borrelia-RN-AT; Euroimmun AG, PerkinElmer, Inc., Waltham, MA, USA). LB was diagnosed and oral cefuroxime was prescribed at the dose of 30 mg/kg body weight/day [5,9]. She was also referred to the hospital for a skin biopsy.

During hospitalization on the infectious diseases ward, the child underwent basic laboratory tests which showed no abnormalities: leucocyte count 4.57 × 10^9^/L; hemoglobin concentration 11.6 g/dL; thrombocyte count 307 × 10^3^/L; CRP 0.02 mg/dL; erythrocyte sedimentation rate 4 mm/h; total serum IgG concentration 1030 mg/dL; rheumatoid factor negative. No anti-nuclear (ANA) or anti-neutrophil (ANCA) antibodies were found in the serum. Using the ELISA test, there were also no confirmed infections with viruses: hepatitis B and C, cytomegalovirus, Epstein-Barr virus. A skin biopsy was performed under local anesthesia. Polymerase chain reaction (PCR) was performed on the skin biopsy specimen (non-standardized test, Laboratory for Molecular and Forensic Genetics, The Ludwik Rydygier Medical University in Bydgoszcz, Poland), but Bb DNA was not detected. Histopathological examination of the skin biopsy section revealed a thin epidermis with vacuolization of the basal layer, and numerous melanophages under the epidermis. In the deeper layers of the skin there were small clusters of lymphocytes surrounded by a dense homogeneous, eosinophilic stroma. The picture corresponded to morphea. Localized scleroderma initiated by Bb was diagnosed. In therapeutic management, antibiotic therapy with oral cefuroxime was continued for 4 weeks [5,9]. The mother was also recommended to continue dermatological treatment, but the parents decided not to follow hospital recommendations.

A control examination 2 months after the end of antibiotic therapy showed local improvement of the skin condition, and relief of pruritus. The area of previous plaque was covered with a dry layer of epidermis without induration (Figure 3B).

## 3. Discussion

### 3.1. Cutaneous Manifestations of Lyme Disease

The occurrence of skin lesions is the earliest symptom of Lyme disease, and the diagnosis of these lesions and appropriate treatment may prevent complications of the disease, which are mainly neurological. The cutaneous manifestation is heterogeneous. There are typical lesions that constitute the basis for the diagnosis of LB, and atypical ones which cause significant diagnostic difficulties, especially when the patient does not remember the tick bite. The three main dermatological symptoms, EM, LC, and acrodermatitis chronica atrophicans (ACA), occur in phases. The disease is also thought to influence the incidence of other dermatological conditions, including scleroderma, lichen sclerosus, and more recently B-cell lymphocytoma [7,8,10].

### 3.2. Tick-Bite without Erythema Migrans

Many parents present their children to the doctor immediately after being bitten by a tick. Some of them look for medical assistance to help remove the tick from the child’s skin. Other parents are concerned about whether self-removal was correct, or whether antibiotics are required post-exposure. The first step is to remove the tick as quickly as possible. It has been proven that the risk of Bb infection increases with the time the tick remains in the patient’s skin. After removing the tick, the bite area should be cleaned with alcohol or soap and water. Serological or molecular tests of the removed ticks are not recommended, as their positive results do not constitute grounds for treating a child.

**Figure 4:**
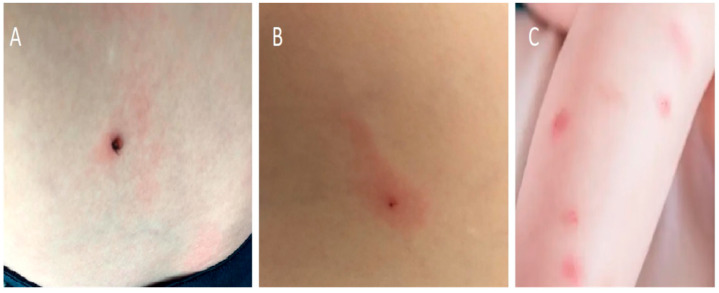
Skin lesions caused by a tick bite without EM—(**A**) skin reaction with a tick in the skin, (**B**) skin reaction after a tick bite, (**C**) skin reaction after a mosquito bite.

Prophylactic administration of an antibiotic to a child bitten by a tick is not recommended practice. The best method of preventing LB in children is considered to be pre-exposure prophylaxis such as protective clothing, tick repellents, checking and removal of ticks after a journey in an endemic zone, and in the case of a tick bite, regular examination of the bite site during the following weeks in order to initiate an early curative treatment if ECM is diagnosed [11]. However, the current Infectious Diseases Society of America (IDSA) recommendations recommend that prophylactic antibiotic therapy be given to adults and children within 72 hours of removal of a high-risk tick bite. A tick bite is considered to be high-risk only if it meets the following three criteria: the tick bite was from (a) an identified *Ixodes* spp. vector species, (b) it occurred in a highly endemic area, and (c) the tick was attached for ≥36 h. A single dose of doxycycline (4.4 mg/kg, maximum 200 mg) is recommended for all ages. Amoxicillin is not recommended for post-exposure prophylaxis because of its short half-life (approximately 1 hour) versus the longer half-life (16–22 h) of doxycycline [12]. The described antibiotic prophylaxis is included in IDSA, not European, recommendations [13]. It is the first recommendation for the pediatric population so far [12]. Such a procedure, in particular an attempt to identify the species of tick, should not delay the treatment of the patient. In European conditions, the basic approach involves instructing parents about the symptoms—which, in the case of *Borrelia* infection, may develop in days/weeks—and recommending careful observation of the child [13,14] (Figure 4).

### 3.3. Erythema Migrans

**Figure 5:**
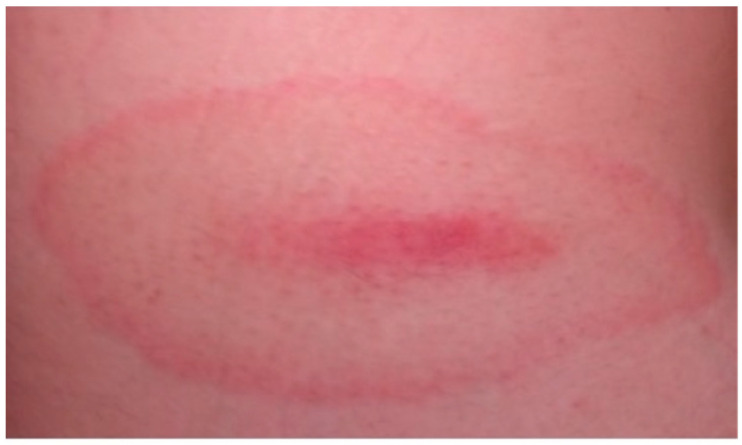
Clasical erythema migrans on the skin of a 6-years-old child.

The classical EM is a spotted, round-shaped rash, sometimes with a raised center that occurs early in infection with *B. burgdorferi*. In 80% of people, it appears on the skin within 3 to 30 days after infection, surrounds the site of the tick bite, and gradually increases in size. According to IDSA, an erythema over 5 cm in diameter is considered pathognomonic [12]. In adults, it occurs most often on the limbs and torso, and in children, on the head and neck, but it rarely affects the skin of the hands, soles of the feet, or mucous membranes [15]. The erythema is usually flat. Its characteristic feature is the occurrence of a dark raised center with a clear ring, and a red band around the outside [16] (Figure 5).

It may be accompanied by general symptoms such as fever, headache, or feeling crushed. In those untreated with antibiotics, EM spontaneously regresses over a period of several days to several weeks (on average within 4 weeks); this does not indicate that the systemic infection has cleared. Atypical forms of EM are irregular in shape, with the presence of petechiae or vesicles, and if they tend to increase in diameter, they should be treated as Lyme disease [17]. EM can be confused with the changes that occur when bitten by insects. The occurrence of typical EM is the basis for the diagnosis of the first stage of Lyme disease, and does not require confirmation by serological tests [9,10,12,13]. The current recommended treatment of children with EM without accompanying neurological and cardiac symptoms includes the early administration of oral antibiotics: doxycycline, amoxicillin, or cefuroxime axetyl [9]. For patients unable to take both doxycycline and beta-lactam antibiotics, the preferred second-line agent is azithromycin [12]. The choice of antibiotic depends on the age of the child, the presence of extracutaneous manifestations of LB, drug allergies, side effect profile, coinfections, and possible sun exposure. In the NICE recommendations, amoxicillin is the first-choice antibiotic for children under 9 years of age. In older children (over 9 years of age), experts recommend treatment with doxycycline, as in adults. Amoxicillin remains the drug of first choice [13]. The implementation of these recommendations is limited by local registrations of doxycycline, which allow the use of the antibiotic over 12 years of age which makes the treatment of younger patients an off-label therapy [5]. The updated Clinical Practice Guidelines by IDSA recommend the use of doxycycline in the therapy of younger children with EM [12]. This recommendation is currently being widely discussed and has both supporters and opponents. The clinical use of tetracyclines in pediatrics is limited due to the known binding of first generation tetracyclines to the teeth and bones of young children, which may result in permanent tooth discoloration and enamel hypoplasia. Doxycycline, which is sometimes referred to as a second-generation tetracycline, has been found to bind calcium less than tetracycline, which may explain why doxycycline may be less likely to cause tooth staining [18,19]. Additionally, doxycycline has the advantage of being efficacious for treatment of human granulocytic ehrlichiosis, which may occur simultaneously with early Lyme disease.

Some experts point out that studies evaluating the safety of doxycycline in children under 4 years of age have not been conducted, nor have there been long-term follow-ups conducted in these patients. Finally, the decision to use doxycycline in young children with LB should be based on assessment of the risk to benefit ratio of the use of this drug compared with that of other recommended antibiotics. The assessment of the total dosage of doxycycline that had previously been taken by a patient should be obligatory, because the risk of dental staining is directly related to cumulative exposure [20].

Current recommendations do not recommend long-term follow-up of children who have completed EM treatment. The clinical outcome after EM antibiotic therapy is good [21].

However, due to the possibility of neurological and cardiac complications in treated patients, parents should be educated on the early recognition of these symptoms [4].

### 3.4. Multiple Erythema Migrans (MEM)

**Figure 6:**
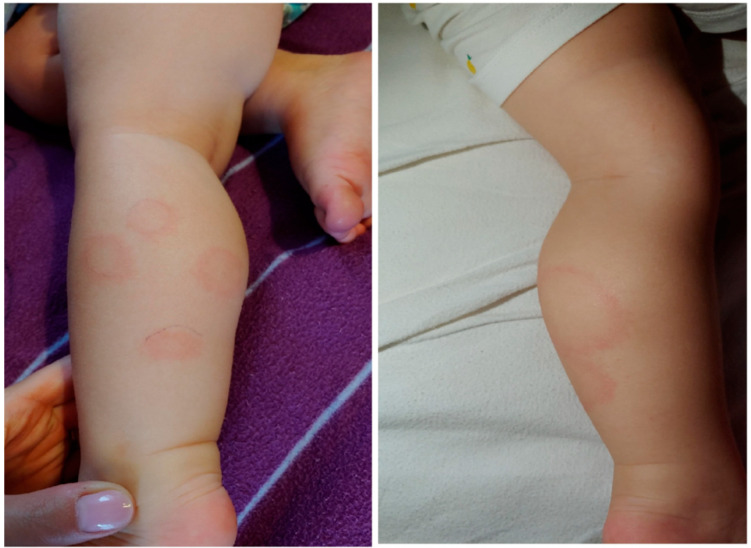
Classical multiple erythema migrans on the skin of a 1-year-old child.

The presence of MEM is evidence of spirochaetemia in the body. It can also be the result of multiple bites on a child. The appearance of MEM may be accompanied by general flu-like symptoms, headaches, joint pain, and neurological symptoms. MEM is a rare manifestation of infection in children [10], and is still often unrecognized as one of the clinical forms of Lyme disease [22]. The location of multiple erythema is not related to the tick bite site. Characteristic erythema appears around the injection site, as in distant areas of the skin [7,23]. If the primary lesion is the erythema around the tick bite, the remaining lesions (usually smaller than the original lesion) are satellite lesions (Figure 6).

The diagnosis of MEM, like EM, is a clinical diagnosis; there are no indications that serological tests should be performed in the presence of lesions of such morphology. Patients presenting with EM and MEM should receive adequate treatment, without delays associated with serological testing. In doubtful cases, the lesions should be observed for the next 24–28 h, and evaluated for peripheral enlargement. Serological tests should be performed at least 2 weeks after the onset of MEM [24]. Patients with early disseminated disease who present with multiple erythema migrans lesions are treated the same way as those with a single erythema migrans lesion [11,12,13].

### 3.5. Acrodermatitis Chronica Atrophicans

The common skin symptom of chronic Lyme disease that does not resolve spontaneously is ACA. Lesions are usually one-sided at first, later becoming more or less symmetrical. Patients with ACA rarely have prior symptoms of Lyme disease, e.g., EM occurs in only 20%. The disease is more common in women than in men, mostly over the age of 40, and is very rare in children [25]. Initially, bluish-reddish discoloration and swelling on the back of the hands, feet, or knees is difficult to see. The lesions enlarge very slowly over the course of months, or even years, then the swelling slowly disappears. The skin becomes thin and wrinkled, purple in color, with visible veins. Healing of skin lesions is slowed down. In some patients, hardened lesions also occur around joints and peripheral nerves [26].

The diagnosis of ACA is based on clinical findings and serological tests (high level of specific *Borrelia* IgG antibodies). IgM antibodies against Bb are not useful for diagnosis. In an uncertain clinical picture, a skin biopsy and histological examination are recommended. Histological examination of late lesions demonstrates an atrophic epidermis, and interstitial lymphocytic infiltrate with plasma cells and occasional histiocytes or mast cells [27]. Detection of BB DNA by culture or polymerase chain reaction (PCR) helps to confirm the diagnosis [28].

### 3.6. Borrelial Lymphocytoma

A rare form of LB, found mainly in Europe, is borrelial lymphocytoma [29]. It appears as a single, painless, bluish-red plaque or nodule located on the ear lobe, auricle, nipple, or scrotum (Figure 2) [30]. It is more common in children than in adults and, in contrast to EM and ACA, has a male predominance. A tick bite has been reported in approximately half of the patients; the tick bite is usually at the site or in the vicinity of the later BL. The most common site for the development of lesions is the breast in adults, and the ear lobe in children [31,32]. The lesion is 1 to 5 cm in diameter and consists mainly of B lymphocytes [31]. It appears on the patient’s body from 1 to 10 months [31] after infection [32]. In the absence of appropriate treatment, the lesion may persist for months, and other manifestations of LB may follow. The diagnosis of BL is based on clinical findings and two-step serologic tests (ELISA and Western blot). At the initial diagnosis, approximately half of the patients with BL have measurable serum borrelial antibodies and, in about one-third, spirochetes can be cultivated from the skin lesion [31]. In some patients, BL is an early symptom of the disease, and therefore there is a risk of a “false negative” result. Based on observations of patients with BL, Maraspin et al. found that a seropositive response to *B. burgdorferi* antigens was found in only 35.3% patients at the initial examination [33]. The children who initially have negative results should be monitored for seroconversion within a short period [33,34]. Histological examination of the skin biopsy from the LB area and immunohistochemistry to define immunophenotype are also suggested (usually CD20 positive, Bcl-2 negative, κ and λ light chain expressed in an equivalent manner, and BB PCR on DNA from skin slides) [8]. Accordingly, Cerroni observation PCR for *Borrelia* on tissue’s DNA (frozen or formalin-fixed and paraffin-embedded) can target OspA [35].

Treatment of the lesion is based on the usual administration of an antibiotic, and the prognosis for resolution depends on the progression of the disease before treatment [9,10]. The case presented above was characterized by a slow recovery. A final consultation showed resolution of the lesion, but the patient required prolonged treatment. It should be mentioned that the etiology of BL also includes other pathogens (including *Treponema pallidum*), viruses (e.g., Herpes virus sp., Molluscipoxvirus, HIV), parasites (e.g., scabies, leishmaniasis), and non-infectious agents (certain drugs and UV radiation).

### 3.7. Localized Scleroderma (Morphea)

Localized scleroderma is a chronic inflammatory disease of the connective tissue with an autoimmune background. There are two high incidence peaks—one in children aged 7–11 years, and the second at age 40 to 50 years in adults. The disease is much more common in women (2.6–6 times more often). About 15% of patients are children under the age of 10. It is estimated that the incidence of morphea is 0.4–2.7/100,000 people per year [36]. It is characterized by an inflammatory phase followed by a process of skin fibrosis leading to thickening and hardening [37]. The main cause of morphea, apart from genetic factors, weakness caused by radiotherapy, and individual tendency to develop autoimmune diseases, is also infection with Bb. Currently, the mechanism of the development of autoimmune processes in people suffering from LB is not fully known. One of the cases presented above was a patient with local scleroderma and high serological indicators of Bb infection. The initiation of treatment typical for LB resulted in the disappearance of skin lesions, which confirms the influence of LB on the occurrence of skin lesions [38]. The importance of LB in the formation of morphea is debated. Presented by different researchers, the prevalence of anti-Lyme antibodies in people with morphea is variable. It ranges from 4–19 percent.

## 4. Conclusions

Although knowledge about the effects of *Borrelia burgdorferi* infection and skin manifestations is increasing, general practitioners continue to overlook the diagnosis of Lyme disease. Borrelia infected ticks exist in large areas of the world and locations include woodland, recreational parks, and even gardens. It is worth emphasizing that skin symptoms are early symptoms of Lyme disease, and can lead to debilitating arthritis, neurological, and cardiac abnormalities in the later stages. Hence, the identification of skin features, including those rarely manifested, may lead to early diagnosis of the disease and prevention of the development of further advanced diseases. Any EM rash is diagnostic for LB and should be treated immediately with the recommended antibiotics. Raising the awareness of the public and family doctors seems to be a step in the right direction to achieve this goal.

An important element of the prevention of long-term consequences is also educating parents about the possibility of their occurrence, and covering small patients after antibiotic treatment with further medical supervision.
